# Experimenting to increase the effectiveness of a national campaign on hygiene behavior in Tanzania

**DOI:** 10.1038/s41598-024-67144-0

**Published:** 2024-07-19

**Authors:** Robert Aunger, Aidan Coville, Lukas Kwezi, Anyitike Mwakitalima, Kaposo Mwambuli, Arndt Reichert, Jérôme Sansonetti

**Affiliations:** 1 Department of Disease Control, London School of Hygiene and Tropical Medicine, London, WC1E 7HT UK; 2 World Bank, DIME, Pretoria, South Africa; 3 Tetra Tech, Iringa, Tanzania; 4 Water, Sanitation and Hygiene Sub Unit, Tanzania Ministry of Health, Dodoma, Tanzania; 5 Project CLEAR, 1297 Dar es Salaam, Tanzania; 6 Leibniz University Hannover, 30167 Hannover, Germany; 7 World Bank, DIME, 75116 Paris, France

**Keywords:** Health care, Risk factors

## Abstract

Through Tanzania’s National Sanitation Campaign, we study the effectiveness of two common elements of behavior change campaigns: endorsements from celebrities and testimonials. Using four experiments in Tanzania in early 2021 as part of the national campaign, we find that including endorsements and testimonials in text messages sent to individuals significantly increases self-reported hygiene behavior. These results mask important heterogeneity based on the source of endorsement or framing of the testimonial and provide insights into cost-effective approaches for changing behavior at scale.

## Introduction

Two million preventable deaths occur yearly around the world due to inadequate Water, Sanitation and Hygiene (WASH) conditions^1^. Sub-Saharan Africa is disproportionately affected, with roughly two thirds of WASH-related under-five deaths globally^2^. Addressing these challenges through WASH interventions has found mixed results^3,4^. Hygiene behaviors such as washing hands with soap have been shown to significantly improve health outcomes^5^ and potentially effective and scalable interventions have been identified^6^. However, WASH-related behaviors have still traditionally been difficult to change through national programs at scale^7–10^. This paper aims to contribute to the behavior change literature by conducting multiple experiments aimed at improving the effectiveness of a national campaign to change WASH-related behaviors at scale.

Our study was designed to support Tanzania’s National Sanitation Campaign (NSC), *“Nyumba ni Choo”*, whose goal is to more than double access rates to basic sanitation and hygiene to 75%. Specifically, we worked alongside the NSC to experimentally trial different behavioral designs. The campaign ran television commercials on eight different TV channels, aired radio spots on 32 different radio channels, and was complemented with additional media (e.g., on-the-ground events, social media engagements, road-shows) as well as materials (e.g., t-shirts and caps). During the pandemic, the campaign switched to COVID-related messaging, signaling that everyone should continue to practice hand washing with soap, face hygiene by using face masks, and physical distancing^11^. It is during this latter phase that randomized behavior interventions were delivered through questions to respondents across the country participating in short message service (SMS) surveys as a way to test and finetune campaign messages.

In this paper, we experimentally examine endorsements and testimonials as strategies aimed at improving the effectiveness of the NSC. Endorsements are provided by celebrities who publicly associate themselves with a campaign and are often stars from music, movies, and sports, as well as famous influencers (see e.g., “goodwill Ambassadors” for the United Nations and its organisations). The theoretical underpinning of endorsements is based on the transfer of cultural meanings from endorsers to campaign objects and subsequently to receivers^12^. In our experiment, we vary whether the celebrity is a sports star, popular model, or famous subject expert. Theses celebrities embody different cultural meanings that align with the campaign’s objectives, and their public personas serve as aspirational figures for receivers, facilitating the integration of these meanings into the receivers’ self-identity and worldview. Testimonials, on the other hand, are life stories of fictional everyday persons, which offer more vivid, personal illustrations of a campaign’s messages. Addressing both cognitive and emotional perspectives^13^, testimonials serve as availability and simulation heuristics, making vividly presented information more likely to be recalled and imagined in future scenarios^14,15^. Vividly presented testimonials evoke emotions like anticipated worry and regret that may lead to behavior changes^16^. We experimentally vary positively and negatively framed testimonials. Motivated by prospect theory, we explore whether people value losses more than gains in this context, which would predict a stronger response from negatively rather than positively framed messaging^15^. However, the opposite response may occur when negative framing is perceived as unfair, causing resistance^17^.

To identify the impact of endorsements and testimonials, we conduct four randomized controlled trials (RCTs), which include seven treatment arms in total. Experiment 1 examines the impact of endorsements by a soccer player on Tanzania’s national team and the Ministry of Health on a handwashing campaign. Experiment 2 tests endorsements for handwashing by the same soccer player and a medical doctor who appears in Tanzanian media as a health commentator at the national level. Experiment 3 compares the effect of the endorsement of sanitation by a Tanzanian model and philanthropist with a testimonial relaying the fictional story of a woman who lost her three-year-old baby following his contamination from fecal matter. In Experiment 4, two treatment arms trial testimonials concerning sanitation which are positively framed and negatively framed variations of the same fictional story. Thus, in total, there are five experimental groups across three experiments (Experiment 1–3) which receive an endorsement treatment and three experimental groups across two experiments (Experiments 3 and 4) that receive a testimonial treatment. Each experiment was self-contained within an SMS survey. Respondents were first asked a set of descriptive questions that were used to test for baseline balance. This was followed by the testimonial or endorsement intervention. A further set of questions were then collected directly after the treatment was administered. They measure water use and interest in improved sanitation and were forward looking so that respondents’ answers could reflect treatment effects on intention for the future. Since different experiments pertain to different topics, these outcomes were pooled and standardized to analyze the overall effect of the behavioral strategies across different settings, enhancing the robustness and comparability of the data. Experiments 1 and 3 were conducted in the first, and experiments 2 and 4 in the second SMS survey.

We find that endorsements cause a significant increase in desired behavior change outcomes. However, this masks important heterogeneity. Specifically, while we observe significant effects for the sports star and the popular model, the effects of the famous subject expert and Ministry of Health are statistically insignificant. In addition, we find that testimonials are, overall, effective at increasing desired behavior change outcomes with negatively-framed testimonials driving the overall effect. There is no statistically significant difference in the effectiveness between endorsements and testimonials.

This paper relates to the marketing and behavioral science literature on endorsements and testimonials. Endorsements have been found to be effective in influencing perceptions about conflict^18^, politics^19^, and public health^20–22^, although the evidence is often descriptive, rather than causal, and seldom compares different forms of endorsement within a common study design. Testimonials have been shown to reduce defensiveness^23^ and increase health awareness^17^. Consistent with insights from prospect theory^24^, negative framing has been shown to be more effective than positive framing, for instance to drive up mammography uptake^25^ or exercise^26^. However, this is not universally the case^27,28^ and the relative effectiveness of positive versus negative framing appears to interact with subject characteristics, for instance to drive up the immunization of infants^29^ or skin cancer detection^27^, leading to ambiguous predictions.

The paper also links to interventions based on health-related text messages sent to the phones of individuals^30^. For instance, a randomized experiment investigates the impact of an SMS-based information campaign on the adoption of social distancing and handwashing practices in rural India during the COVID-19 pandemic^31^. While it similarly compares positively and negatively framed messages, the campaign used neither endorsements nor testimonials. In addition, it connects with literature experimentally evaluating programs aimed at promoting hygiene behavior in Tanzania, although it does not specifically focus on endorsements or testimonials as our paper does^9,32^.

We contribute to this literature in three ways. First, we expand the limited evidence base on understanding how behaviors known to be important can be affected at scale through national campaigns^22^ and low-cost SMS experimentation^31^. We add a novel perspective by sending endorsements and testimonials concerning hygiene behavior via text messages. Second, by compiling seven treatment arms, we are able to explore heterogeneity in the design of each of the strategies to strengthen our understanding of potential underlying drivers linked to commonly-used behavior change interventions. Third, by testing the behavior change strategies in the same study, we contrast their respective merits for policy applications.

## Methods

### Experiments

To assess the effectiveness of endorsements and testimonials, we conduct four RCTs. They compile a control group and seven treatment arms. The experiments are embedded in two original SMS surveys collected as part of this study: one of the survey questions is used to inform respondents, and depending on the randomized subgroup, that information treatment differs. All treatment subgroups use the same base campaign, and the treatment arms are variations from this.

The four experiments conducted in this study are briefly described here, and the details are shown in-full in Table 1.

- Experiment 1 - *Endorsement variations*: The control group receives a base campaign message concerning handwashing, while the first treatment arm pairs the base campaign message with an endorsement by a soccer player on Tanzania’s national team, and the second treatment arm pairs the base campaign with an endorsement by Tanzania’s Ministry of Health.
- Experiment 2 - *Endorsement variations*: The control group receives the same base campaign message, while the first treatment uses the endorsement of the same soccer player, and the other the endorsement of a medical doctor who appears in Tanzanian media as a health commentator.
- Experiment 3 - *Endorsements and testimonials*: The control group is not shown any base campaign message, while the first treatment arm informs participants that sanitation is endorsed by a Tanzanian model and philanthropist, and the second treatment arm trials a testimonial relaying the fictional story of Devota, a woman who lost her three-year-old baby Julius following his contamination from fecal matter.
- Experiment 4 - *Positively and negatively framed testimonials*: The control group is not shown any base campaign message, while the two treatment arms trial testimonials which are variations of Devota’s fictional story described above.

Institutional review board approval was received from Solutions IRB (https://www.solutionsirb.com/;Protocol#2020/08/32) and all procedures were performed in accordance with this protocol, including receiving informed consent from all participants.

### Outcomes

Treatment effects are measured along outcomes which are self-reported, and collected in the survey where experiments are embedded, after treatments are administered. This study draws on three outcome variables in total, shown in-full in Table 2. The first outcome measures respondents’ use of water (*water use*). For this measure, respondents were asked to allocate a fixed amount of water to handwashing, at the expense of other water needs. The second outcome, *sanitation investment*, measures investment into sanitation by capturing the share of a fixed grant amount that respondents allocate to toilet, when the alternative investment categories are water and electricity. The third outcome measures the respondent’s priority of sanitation (*sanitation priority*), which indicates whether the respondent perceives having an improved toilet to be the most important way to keep children healthy. Regarding the two continuous outcome variables concerning water use and sanitation investment, the survey questions are worded in a forward-looking manner, so that respondents’ answers can capture treatment effects. This means that we asked respondents how they intend to act in the near future, rather than how they acted in the past. We recode outcomes so that they can be used in regressions, in the way shown in the last column of Table 2. As a convention throughout this paper, all outcomes are coded in ascending order of desirability.

Not all outcomes are asked in each experiment, rather we ask the outcomes corresponding with the WASH topic of the experiment. This includes one outcome for handwashing experiments (*water allocation*) and two outcomes for sanitation (*sanitation investment* and *sanitation priority*). In Supplementary Figs. S1–S4, we provide one diagram for each experiment, showing the treatment subgroups alongside the outcome numbers used to measure effects.

### Data and sampling

This study draws on original data from two survey rounds collected in January 2021 (Survey #1) and June 2021 (Survey #2) with more than 1,000 respondents each. The surveys were administered in Swahili, Tanzania’s national language, and their observation unit is the individual. Almost two hundred individuals participated in several SMS surveys which included experiments that we conducted since the start of the pandemic (one survey in summer 2020 without experimentation using endorsements or testimonials in addition to the two surveys presented in the present paper). To focus on unique observations, their surplus observations were dropped. After those individuals are removed, the sample size of each survey is 1,279 for Survey #1 and 1,122 for Survey #2, yielding a total of 2,238 unique individuals. As a robustness check, we also run a model where surplus observations are left in (see below).

The surveys were collected by self-fill SMS. Self-fill SMS is a collection mode where questions and responses are administered by text, via the respondents’ phones. The survey starts with an invitation to take part in the survey. If respondents provide consent, they receive the survey’s questions one by one, and respond by text their answers. Depending on the answers they text, the flow of questions adjusts, following patterns programmed ex-ante. Answering the survey is free of charge to the respondents, as the costs are paid by the survey firm. Respondents who complete the survey receive airtime, sent to the phone number they used to take the survey within two days. The gross amount of airtime is TZS 1,000, inclusive of TZS 153 VAT, i.e., a net airtime amount of TZS 847, equal to USD 0.36 at the time of writing. This amount of compensation is in line with local customs: high enough to show appreciation, while not excessive.

The sample frame for these surveys starts from lists of sim card users registered with the three largest phone operators in Tanzania: Vodacom, Airtel, and Tigo. At the beginning of every year, the survey firms conduct a process called “users indexing”, where phone users are approached and asked if they would agree to take part in future SMS surveys. Those who agree eventually form the sample frame used to build the sample. Sampled phone numbers receive a text offering for them to participate. Those who opt to do so enter the survey. To support representativeness, the sample is built to match national-level statistics along four variables (region, rural vs urban, gender, and age groups), whose targets are provided in Supplementary Table S1. The target sample size is 1,000 respondents by survey, however as a result of filling these quotas, the final sample size slightly exceeds 1,000. Supplementary Tables S2 and S3 display descriptive statistics for the study population in each survey. A step-by-step guide describing the study implementation is provided in the Supplementary Information.

It should be noted that when several experiments are conducted in the same survey, the respondents from that survey participate in all those experiments. For instance, Experiments 1 and 3 were both conducted in Survey #1. Therefore, by the time respondents participate in Experiment 3, they have already received the treatments from Experiment 1. Importantly, random assignments across experiments are independent from each other. Thus, treatment status from earlier experiments is expected to be balanced in later ones, and therefore not to drive the results of later experiments.

### Balance

The baseline balance tables for the experiments are shown in Supplementary Tables S4–S9, and we confirm that randomization ensured balance across observed measures. For instance, Supplementary Table S6 assesses balance for Experiment 3. It includes 13 pre-intervention covariates – for each, the table shows the means of subgroups, the mean differences across subgroups, and their statistical significance. With three subgroups, 13 covariates result in 39 mean comparisons, for which the number of expected “by-chance” imbalanced tests at the 5% level is 1.95. We find one imbalanced test significant at the 5% level, and four at the 10% level, with small magnitudes.

Because this paper analyzes results by pooling observations, below we also report balance at the pooled level. To allay concerns that the existing imbalances could partly drive the findings, we further run a model where all covariates found to be imbalanced at 10% in any of the experiments are included as controls. We discuss this as a robustness check in the below subsection, and find that the results are not driven by these imbalances.

### Empirical strategy

Randomized assignment of the interventions is secured based on a cryptographic random number generator, using the cryptographic service provider. This assigns respondents to the various subgroups randomly following a uniform distribution. Random assignment is done across all SMS enumerations which are active at a given point in time. We do not anticipate spillovers between participants, as they are spread across the sample’s region, and are not connected to each other.

To organize findings on the behavioral designs trialed in this study, we pool the seven treatment arms into endorsements and testimonials. This section starts by describing how the observations are pooled, and then presents the econometric specification.

#### Pooling of treatment

We start by appending the data sets from the two surveys. Then, for the two behavioral change strategies examined in this paper, we build an overall pooled treatment variable. For instance, five treatment arms in this study relate to endorsements, and the pooled treatment variable is equal to 1 if the observation belongs to any of those, and 0 otherwise. Second, we build “subpool” treatment variables, where we pool together only some of these treatment arms, based on a given characteristic they share. For instance, within the pooled analysis of endorsements, we build a subpool treatment variable for all endorsements by celebrities, and one for those by non-celebrities. This allows us to explore different levels of heterogeneity in treatment. One particular case is when the pooled analysis of a behavioral design draws on several experiments coming from the same survey. For instance, the pooled analysis of endorsements draws on the treatment arms from Experiment 1, and the first treatment arm from Experiment 3. It is not immediately possible to generate the overall pooled treatment variable, as a given individual from Survey #1 may be in control under Experiment 1, and in treatment under Experiment 3. In this case, the data include for the same individual two observations. One observation corresponds to the treatment assignment and outcome variable in the first experiment and the other corresponds to the treatment assignment and outcome variable in the third experiment. The econometric specification controls for the experiment that generated the observation (see Equation 1).

For endorsement, we draw on a total of five treatment arms from three experiments conducted across two surveys. The details of these treatment arms are shown in Supplementary Fig. S5. They include endorsements by Tanzania’s Ministry of Health and by three celebrities: a soccer player on Tanzania’s national team; a fashion model and philanthropist; and a medical doctor providing health commentary in Tanzanian media.

The pooled analysis of testimonials draws on three treatment arms from two experiments conducted across Survey #1 and Survey #2, and the details are shown in Supplementary Fig. S6. The three treatment arms relay the fictional story of an everyday person named Devota and her three-month-old son Julius. Two treatment arms (Testimonials #1 and #3) explain that Julius died from dehydration, due to lack of an improved toilet in the household. In contrast, the third treatment arm (Testimonial #2) explains that Julius is in good health, and that the household benefits from having an improved toilet.

#### Pooling of outcomes

We pool experiments which sometimes use different outcomes to measure effects. This occurs primarily when the experiments being pooled pertain to different WASH topics (handwashing, clean water, etc.). Specifically, for the pooled endorsement analysis, we draw from experiments which relate to handwashing (all using one same outcome, *water use*) and one treatment arm relates to sanitation (using two outcomes, *sanitation investment* and *sanitation priority*). As all testimonial arms relate to the topic of improved toilets, they use the same two respective outcomes, i.e., *sanitation investment* and *sanitation priority*.

To support comparability across experiments, we produce pooled outcomes for each pooled treatment analysis, i.e., one for endorsements (*index of handwashing and sanitation*) and one for testimonials (*index of improved sanitation*). Following the approach of standardized mean differences commonly found in meta-analyses^33^, we build pooled outcomes in two steps. First, we recode the outcomes obtained in a given experiment from 0 to 1 and average the various recoded outcomes into an experiment index. Second, we standardize the experiment’s index with mean equal to 0 and standard deviation equal to 1 in the experiment’s control group. For instance, we asked respondents receiving the endorsement of improved toilets provided by the model the questions in relation to the outcomes *sanitation investment* and *sanitation priority*. We calculate the average over the two re-coded outcomes and standardize this pooled variable.

The details of these calculations are provided at the bottom of the diagrams detailing the pooling procedure in Supplementary Figs. S5 and S6.

### Econometric specification

The pooled analyses of the behavioral designs are conducted using the econometric specifications described below. Here, we describe the specification using endorsements as an illustration, but the same specification applies to testimonials. We probe the robustness of the results with respect to clustering the standard errors by individuals and including imbalanced baseline covariates. Across the study, we declare as survey design in Stata that the sample is stratified by region.

Equation 1 specifies the model for average treatment effects of endorsements, using the terms below.

1 $$\begin{aligned} Y_{i}=\alpha _0+\sum _{j=1}^{J}\alpha _jEndorsement_{ij}+\sum _{k=1}^{K}\beta _kExp_{ik}+\varepsilon _{i} \end{aligned}$$

- $Y_{i}$ is the outcome variable for individual *i*.
- $Endorsement_{ij}$ is the treatment variable for an endorsement of type *j*, equal to 1 if individual *i* was treated with it, and 0 otherwise. For instance, if the model includes a subpool of all endorsement arms by celebrities and a subpool of all endorsement arms by non-celebrities, we include $Endorsement_{i1}$ as the treatment variable for the former, and $Endorsement_{i2}$ as the treatment variable for the latter.
- $\alpha _j$ is the coefficient of interest for endorsement of type *j*.
- $Exp_{ik}$ is an indicator equal to 1 if individual *i* received experiment *k*, and 0 otherwise. As individual experiments are self-contained within each survey round, i.e., specific treatments are solely provided in one of the two survey rounds, the binary variables indicating the experiment simultaneously control for the survey the observation was collected in. For example, the first and third experiments were both conducted within the first survey round, which is why respective binary indicators additionally capture fixed effects pertaining to this survey round.

The set of observations starts from the dataset appending the two surveys. Each respondent participated in one of the two survey rounds. We drop from the dataset respondents who did not participate in any experiment related to the behavioral design at hand.

We run two additional models as robustness checks. First, we add to Eq. (1) a vector of covariates $X_{i}$ found to be imbalanced across subgroups. Second, we add regional fixed-effects to Eq. (1). The results are presented in the additional model section in the Supplementary Information (Tables S10–S17).

## Results

**Figure 1:**
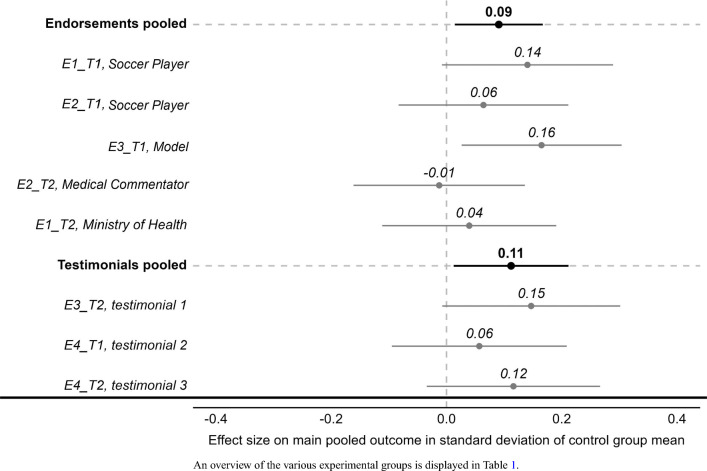
Treatment effect for every treatment arm.

We start with the presentation of the results of the two behavioral strategies on the pooled outcomes (Fig. 1). First, for each of the treatment arms, we show the effect on the main pooled outcome in terms of percent of the control groups mean along with a 95% confidence interval. Second, for each behavioral design, we pool all relevant treatment arms and display the overall treatment effect in the graph. Our results at the overall pooled level reveal that both endorsements and testimonials are effective at increasing desired WASH behaviors.

### Endorsements

Here we study the overall effect when pooling all five endorsement treatment arms together. We find that endorsements cause a significant positive effect on the pooled outcome variable capturing WASH beaviors (Column 1 of Table 3). When we restrict to the handwashing outcomes (Column 5), the overall effect of endorsements is not found to be significant.

We then differentiate between different types of endorsements, to contrast their effectiveness. We build a subpool gathering the four endorsement arms provided by celebrities, therefore excluding the Ministry of Health. We find that the overall effect is driven by endorsements by celebrities, as the subpool coefficient in Column 2 increases compared with Column 1, while the Ministry’s treatment arm is insignificant. One possible explanation is that only the former endorsements transfer cultural meanings onto the campaign’s object. Celebrities can help the campaign’s object acquire the meanings they embody, which then inspires receivers to want to adopt them. In contrast, while the Ministry of Health can disseminate information, it may be less likely to embody cultural meanings.

We build another subpool gathering only endorsement arms from the soccer player and the model, excluding the health commentator. The former two drive the overall effect, while the inclusion of the health commentator has no observable impact. This is reflected in Column 3 of Table 3. Further, Column 4 shows that each of the soccer player’s and model’s endorsements is effective. For this same subpool, we observe a significant effect on the handwashing outcome (Column 8). Several factors may be driving this difference – we review some of them here, although we are unable to confirm them causally or adjudicate between them. One possible factor is that the two effective endorsers are more famous than the health commentator. To explore this descriptively, we review their numbers of Instagram followers as of January 2023. The model has the most followers (1.8 million) and the medical commentator has over twice as many as the soccer star (0.5 million vs 0.2 million). Another possible factor is that participants may view the two effective endorsers more favorably. We explore this descriptively, as Experiment 2 collected additional outcomes measuring the endorsers’ approval ratings. Respondents were given multiple choices (i.e., high approval, medium approval, low approval), which we convert into an index ranging from 0 to 1. We restrict ourselves to the control group of Experiment 2, to neutralize treatment effects, and find that the mean approval indices of the soccer player (0.72) and the medical commentator (0.71) are not significantly different from each other.

It is noteworthy that the medical commentator is the only one of the three celebrities with a medical degree, yet his endorsement is not found effective even though the campaign relates to health. In contrast, endorsements by a soccer player and a model are found to be effective. This seems to align with the notion that an endorser’s effectiveness is not driven by their private expertise, but rather by the alignment between the campaign and the cultural meanings embodied by their public persona.

### Testimonials

When we pool together all three testimonial treatment arms, we find that they cause a significant increase in the main pooled outcome (Table 4, Column 1). This is in line with the notion of testimonials helping individuals to recall provided information more easily and faster through an availability heuristic^14^, or to imagine and construct scenarios through a simulation heuristic^15^.

When distinguishing between the two outcomes, the effect is found specifically for the outcome surveying whether the respondents prioritize sanitation (Column 5). In contrast, no effect is found on the outcome measuring the respondent’s willingness to pay for improved toilets (Column 3).

When differentiating between the treatment arms being pooled, we observe that two of the three testimonials are driving the overall effect. Testimonial #1 causes a positive increase in both the index and the individual outcome *sanitation priority*. Testimonial #3 causes a positive increase in the latter only. In contrast, we find no effects of Testimonial #2. Testimonials #1 and #3 have in common that they were both negatively framed, while Testimonial #2 is positively framed. This finding is consistent with predictions from prospect theory^24^, which assumes that individuals weigh losses (the tragic death of the child) more than gains (the successful survival of the child).

This suggest that testimonials may operate through presenting information vividly. This can help individuals recall the information more easily and faster, through an availability heuristic^14^, or help them imagine and construct scenarios, through a simulation heuristic^15^. Also, vividly presented information causes receivers to feel emotions, which can play a part in decision-making, and stimulate behavior change. Indeed, shocking information may be more likely to generate anticipated feelings among receivers, such as worry and regret.

### Endorsements versus testimonials

To examine which of endorsements and testimonials are more effective, we first compare the results of the pooled analysis. The results displayed in Fig. 1 indicate that the two pooled treatments each caused a significant increase in the pooled outcome, and when compared with each other, the effects do not significantly differ. Second, we leverage that this study comprises one experiment which had a treatment arm for each of the behavior change strategies, i.e., Experiment 3, directly contrasting endorsements and testimonials. The results of this experiment largely confirm the results from the pooled analyses.

Specifically, we find that both treatments have a positive effect on *sanitation priority*. For *sanitation investment*, only the effect of the endorsement is statistically significant. However, the point estimates do not significantly differ from each other in either regression (Columns 1 and 2 of Table 5). This suggests that the two behavior change strategies are equally effective, although, given the cost associated with securing an endorsement, this would suggest that testimonials may be a more cost-effective intervention.

### Quantitative interpretation

In this subsection, we aim to provide a quantitative interpretation for the most promising interventions, i.e., endorsements of the model and the soccer player as well as the negatively framed testimonial. For that, we compare each of the three interventions with the control group for the outcomes the respective messages relate to. To support the interpretation of the results, we use the original (i.e., non-standardized) outcomes variables at the expense of comparability across the three interventions and the various outcome variables. The results are displayed in Table 5.

We observe that the model’s endorsement (first testimonial) increases investments in an improved toilet by 0.25 lakis (0.042 lakis), which corresponds to an increase of 3.5 percent (0.6 percent) of the total disposable budget that is allocated to sanitation. The likelihood of reporting an improved toilet to be the most important way of keeping children healthy raises by 5 percentage points from the endorsement and 6 percentage points from the first testimonial. When the household is able to use 20 liters of water a day, the soccer player’s endorsement increases the amount of daily water used for handwashing by almost one liter or 5 percent.

### Discussion

Governments, non-governmental organizations, and the private sector invest billions each year into campaigns aimed at changing behavior. While focus groups, qualitative evidence, or descriptive quantitative research are often used to inform these designs ex ante, this study highlights the important and powerful role low-cost pilot experimentation can play in helping inform the success of national campaigns. Specifically, the results from the current experiments largely function as proof of concept, indicating that endorsements and testimonials can be effective elements of hygiene behavior change programs. However, the relevance of these findings for other settings should be contextualized within the study’s technical limitations. First, sharing information through text messages may not fully exploit the potential of endorsements and testimonials, which could be even more effective in alternative media (e.g., TV, radio, print) with higher salience. Second, the outcomes measure participants’ self-reported intentional behavior, which may differ from actual hygiene behaviors^9^. Third, SMS surveys are constrained by character limits and lack of interaction with enumerators. This survey technique tends to over-sample better-off respondents who are literate and have access to phones. This does not affect the internal validity of the experiments (which were randomized within participants that opted in to the study) but does affect the potential external validity of the results to the broader Tanzanian population. Also, the effectiveness of an intervention in one setting is not automatically transferable to other contexts. We therefore regard our results as important pointers to policymakers in favor of adopting a similar strategy in a pilot that is experimentally accompanied with the objective of informing scale-up decisions.

## Conclusion

This study evaluates the impact of endorsements and testimonials as behavioral change strategies in the context of a large, national campaign in Tanzania. We do this through four randomized controlled trials with a total of seven treatment arms. In these experiments, we measure effects on self-reported behavioral intentions. The RCTs were embedded in original SMS surveys collected in 2021 in mainland Tanzania.

We find that, overall, endorsements and testimonials cause a significant increase in desired self-reported WASH outcomes. While the two behavior change strategies are similarly effective, we show that the specific design of the treatments matters. We find that the overall effect of endorsements is driven by celebrities rather than famous subject experts or government authorities. This suggests that celebrities may help the campaign’s object acquire the meanings they embody, which then inspires receivers to want to adopt them. Among the tested testimonial variants, those with a negative frame are found to yield stronger results, consistent with prospect theory.

This study helps document that simple, low-cost behavioral designs exist that have the potential to drive up WASH behavior at scale. It also sheds light on key features that influence these effects and point to practical ways to increase the potential effectiveness of at-scale behavior change campaigns, and provide an important complement to WASH infrastructure investments.

**Table 1:** Experiments and treatment subgroups.

Experiment number	Behavioral design	WASH topic	Treatment subgroup		Treatment priming
Experiment 1	Endorsement	Handwashing	E1_C	Control	Information: “Washing hands regularly saves lives”
			E1_T1	Soccer player	Information: “[Soccer player]” said: ‘Washing hands regularly saves lives”
			E1_T2	Ministry of health	Information: “Tanzania’s Ministry of Health said: ‘Washing hands regularly saves lives’”
Experiment 2	Endorsement	Handwashing	E2_C	Control	Information: “Washing hands regularly saves lives”
			E2_T1	Soccer player	Information: “Tanzanian soccer player [soccer player] said: ‘Washing hands regularly saves lives’”
			E2_T2	Medical commentator	Information: “Tanzanian Doctor [medical commentator] said: ‘Washing hands regularly saves lives’”
Experiment 3	Endorsement testimonial	Sanitation	E3_T1	Model	Tanzanian celebrity model. Through her charity foundation, she supports improved toilets
			E3_T2	Testimonial #1	Devota lost her 3-month old baby Julius to dehydration following his infection from fecal matter. Since then, she supports improved toilets.
Experiment 4	Testimonial	Sanitation	E4_T1	Testimonial #2	Devota’s 3-month old baby Julius has been safe from infections with fecal matter. She thanks having an improved toilet for that.
			E4_T2	Testimonial #3	Devota’s 3-month old baby Julius died of dehydration following his infection with fecal matter. She blames his death on lack of improved toilet.

**Table 2:** Outcomes.

WASH topic	Outcome name (Number)	Outcome as surveyed	Outcome recoded
Handwashing	Water use (Outc_1.a)	Imagine tomorrow your household only has 20 liters of water for all needs (i.e. cooking, washing). How many liters will you allocate to handwashing? Reply number between 0 and 20	Number of liters
Sanitation	Sanitation investment (Outc_2.a)	Suppose that next month your household receives a grant of 7 lakis (around $43) which you can allocate between improved toilet, water, and electricity. How many lakis would you allocate to toilet?	Number of lakis
	Sanitation priority (Outc_2.b)	Of these, what is the most important way to keep children healthy?	Dummy equal to 1 if answered a.
		a. Have an improved toilet;	
		b. Give them nutritious food;	
		c. Have access to a health centre

**Table 3:** Pooled effects of endorsements.

	Index of handwashing and sanitation				Water use (liters)			
	(1)	(2)	(3)	(4)	(5)	(6)	(7)	(8)
Pool, all endorsement arms	0.091$$^{**}$$				0.059			
	(0.020)				(0.209)			
Subpool, arms with celebrity endorsement		0.105$$^{***}$$				0.075		
		(0.010)				(0.128)		
Subpool, arms with non-expert celebrities			0.125$$^{***}$$				0.102$$^{*}$$	
			(0.003)				(0.056)	
Subpool, arms with soccer player				0.102$^{*}$				0.102$^{*}$
				(0.056)				(0.056)
Model				0.165$$^{**}$$				
				(0.020)				
Medical commentator			0.018	0.006			0.006	0.006
			(0.795)	(0.927)			(0.927)	(0.927)
Ministry of health		0.020	0.031	0.019		0.005	0.019	0.019
		(0.767)	(0.654)	(0.790)		(0.946)	(0.790)	(0.790)
Constant	$-$0.043	$-$0.052	$-$0.031	$-$0.019	$-$0.021	$-$0.032	$-$0.019	$-$0.019
	(0.287)	(0.203)	(0.479)	(0.679)	(0.625)	(0.471)	(0.679)	(0.679)
Observations	3092	3092	3092	3092	2238	2238	2238	2238

This model pools five treatment arms on endorsements conducted in 2021.

The index of handwashing and sanitation (Pool_Outc_Endorse) is standardized (see Supplementary Fig. S5).

*Water use* (Pool_Outc_1.a) pools information across experiments and is standardized (see Supplementary Fig. S5).

Controls for wave and experiment not shown, base levels not shown * 0.10 ** 0.05 *** 0.01, *p*-val in parentheses.

**Table 4:** Pooled effects of testimonials.

	Index of improved sanitation		Sanitation investment		Sanitation priority	
	(1)	(2)	(3)	(4)	(5)	(6)
Pool, all testimonials arms	0.112$$^{**}$$		0.000		0.145$$^{***}$$	
	(0.028)		(0.994)		(0.004)	
Testimonial 1		0.147$^{*}$		0.045		0.154$^{*}$
		(0.062)		(0.550)		(0.052)
Testimonial 2		0.057		0.019		0.057
		(0.462)		(0.793)		(0.464)
Testimonial 3		0.116		$$-$$0.081		0.219$$^{***}$$
		(0.130)		(0.270)		(0.004)
Constant	$-$0.017	$-$0.000	$-$0.021	0.000	$-$0.004	0.000
	(0.719)	(1.000)	(0.630)	(1.000)	(0.934)	(1.000)
Observations	1840	1840	1840	1840	1840	1840

This model pools three treatment arms on testimonials conducted in 2021.

The index of improved toilet (Pool_Outc_Testim) is standardized (see Supplementary Fig. S6).

*Sanitation investment* (Pool_Outc_2.a) pools information across experiments and is standardized (see Supplementary Fig. S6).

*Sanitation priority* (Pool_Outc_2.b) pools information across experiments and is standardized (see Supplementary Fig. S6).

Controls for wave and experiment not shown, base levels not shown * 0.10 ** 0.05 *** 0.01, *p*-val in parentheses.

**Table 5:** Experiment 3 – Quantitative interpretation of effects of endorsements and testimonials.

	Sanitation investment	Sanitation priority	Water use
	(1)	(2)	(3)
Model (endorsement)	0.251$^{*}$	0.049$^{*}$	
	(0.069)	(0.096)	
Testimonial 1	0.042	0.061$$^{**}$$	
	(0.765)	(0.042)	
Soccer player (endorsement)			0.923$^{*}$
			(0.051)
Ministry of health (endorsement)			0.613
			(0.201)
Constant	3.961$$^{***}$$	0.212$$^{***}$$	8.214$$^{***}$$
	(0.000)	(0.000)	(0.000)
Observations	1279	1279	1279

Non-standardized outcomes are used. Controls for wave and experiment not shown, base levels not show.

$^{+}$ 0.10 $^{++}$ 0.05 $^{+++}$ 0.01 indicate significance level of difference in the coefficients of the two treatments.

### Supplementary Information

Supplementary Information.

## Data Availability

The datasets generated and analysed during the current study are not publicly available due to the inclusion of sensitive information about health-related behavior of participants but are available from the corresponding author on reasonable request.

## Code availability

The code is accessible for replication purpose upon reasonable request to the corresponding author.
