# Author Correction: Experimenting to increase the effectiveness of a national campaign on hygiene behavior in Tanzania

**DOI:** 10.1038/s41598-024-70359-w

**Published:** 2024-08-22

**Authors:** Robert Aunger, Aidan Coville, Lukas Kwezi, Anyitike Mwakitalima, Kaposo Mwambuli, Arndt Reichert, Jérôme Sansonetti

**Affiliations:** 1 Department of Disease Control, London School of Hygiene and Tropical Medicine, London, WC1E 7HT UK; 2 World Bank, DIME, Pretoria, South Africa; 3 Tetra Tech, Iringa, Tanzania; 4 Water, Sanitation and Hygiene Sub Unit, Tanzania Ministry of Health, Dodoma, Tanzania; 5 Project CLEAR, 1297 Dar es Salaam, Tanzania; 6 Leibniz University Hannover, 30167 Hannover, Germany; 7 World Bank, DIME, 75116 Paris, France

Correction to: *Scientific Reports* 10.1038/s41598-024-67144-0, published online 19 July 2024

The Disclaimer section in the original version of this Article was omitted. The Disclaimer section now reads:

The findings, interpretations, and conclusions expressed in this paper are entirely those of the authors. They do not necessarily represent the views of the World Bank and its affiliated organizations, or those of the Executive Directors of the World Bank or the governments they represent.

The original Article has been corrected.

