# Supplementary material for: Experimenting to increase the effectiveness of a national campaign on hygiene behavior in Tanzania
Source: Sci Rep. 2024 Jul 19;14:16703. doi: 10.1038/s41598-024-67144-0 (PMC11271518; doi:10.1038/s41598-024-67144-0)
Supplement: Supplementary file 1 — Supplementary Information. [file 41598_2024_67144_MOESM1_ESM.pdf]

Supplementary Information

Experiment Design

Figure S1. Experiment 1 – Endorsements

Treatment	Information	Outc_1.a
E1_C	Information: *"Washing hands regularly saves lives"*	Imagine tomorrow your household only has 20 liters of water for all needs (i.e. cooking, washing). How many liters will you allocate to handwashing? *Reply number between 0 and 20* ➔ *Number of liters*
E1_T1 SOCCER PLAYER ENDORSEMENT	Information: "Soccer player said: *'Washing hands regularly saves lives'*"	
E1_T2 MINISTRY ENDORSEMENT	Information: "Tanzania's Ministry of Health said: *'Washing hands regularly saves lives'*"	

Figure S2. Experiment 2 – Endorsements

Treatment	Information	Outc_1.a
E2_C	Information: *"Washing hands regularly saves lives"*	Imagine tomorrow your household only has 20 liters of water for all needs (i.e. cooking, washing). How many liters will you allocate to handwashing? *Reply number between 0 and 20* ➔ *Number of liters*
E2_T1 SOCCER PLAYER ENDORSEMENT	Information: "Soccer player said: *'Washing hands regularly saves lives'*"	
E2_T2 MEDICAL COMMENTATOR ENDORSEMENT	Information: "Medical commentator said: *'Washing hands regularly saves lives'*"	

Figure S3. Experiment 3 – Endorsements and Testimonials

Treatment	Information	Outc_2.a	Outc_2.b
E3_C	*NA*	Suppose that next month your household receives a grant of 7 lakis* which you can allocate between improved toilet, water and electricity. How many lakis would you allocate to toilet? ** 1 laki equals TZS100 000, around $43* ➔ *Number of lakis*	Of these, what is the most important way to keep children healthy? a. Have an improved toilet; b. Give them nutritious food; c. Have access to a health centre ➔ *1 = answered a.*
E3_T1 MODEL ENDORSEMENT	Model is a Tanzanian celebrity model. Through her charity foundation, she supports improved toilets		
E3_T2 TESTIMONIAL #1	Devota lost her 3-month old baby Julius to dehydration following his infection from fecal matter. Since then, she supports improved toilets.		

Figure S4. Experiment 4 – Testimonials

Arm	Information	Outc_2.a	Outc_2.b
E4_C	*NA*		
E4_T1 TESTIMONIAL #2	Devota's 3-month old baby Julius has been safe from infections with fecal matter. She thanks having an improved toilet for that.		
E4_T2 TESTIMONIAL #3	Devota's 3-month old baby Julius died of dehydration following his infection with fecal matter. She blames his death on lack of improved toilet.		

Outc_2.a: Suppose that next month your household receives a grant of 7 lakis* which you can allocate between improved toilet, water and electricity. How many lakis would you allocate to toilet?

** 1 laki equals TZS100 000, around $43*

➔ *Number of lakis*

Outc_2.b: Of these, what is the most important way to keep children healthy?

a. Have an improved toilet;
b. Give them nutritious food;
c. Have access to a health centre

➔ *1 = answered a.*

Figure S5. Endorsements Pooled Analysis

Arm	Information	Outc_1.a S1	Outc_1.a S2	Outc_2.a S1	Outc_2.a S2	Outc_2.b S1	Outc_2.b S2
CONTROL	Information: "*Washing hands regularly saves lives*"	X	X				
CONTROL	NA			X		X	
E1_T1 CELEBRITY SOCCER PLAYER	Information: "Soccer player said: '*Washing hands regularly saves lives*'	X					
E2_T1 CELEBRITY SOCCER PLAYER	Information: "Soccer player said: '*Washing hands regularly saves lives*'		X				
E3_T1 CELEBRITY MODEL	Model is a Tanzanian celebrity model. Through her charity foundation, she supports improved toilets			X		X	
E2_T2 CELEBRITY MEDICAL COMMENTATOR	Information: "Medical commentator said: '*Washing hands regularly saves lives*'		X				
E1_T2 MINISTRY OF HEALTH	Information: "Tanzania's Ministry of Health says said: '*Washing hands regularly saves lives*'	X					

Outc_1.a: Imagine tomorrow your household only has 20 liters of water for all needs (i.e. cooking, washing). How many liters will you allocate to handwashing? *Reply number between 0 and 20*

➔ *Number of liters*

Outc_2.a: Suppose that next month your household receives a grant of 7 lakis* which you can allocate between improved toilet, water and electricity. How many lakis would you allocate to toilet?

** 1 laki = $43*

➔ *Number of lakis*

Outc_2.b: Of these, what is the most important way to keep children healthy?

i. Have an improved toilet;
ii. Give them nutritious food;
iii. Have access to a health centre

➔ *1 = answered i.*

$$\text{Pool\_Outc\_Endorse} = \text{Outc\_1.a}_{\text{sdized}}\ (\text{S1}|\text{S2})\ \textit{OR}\ \text{Index\_S1}_{\text{sdized}}(\text{Outc\_2.a}_{\text{sdized}},\ \text{Outc\_2.b}_{\text{sdized}})$$

$$\text{Pool\_Outc\_Endorse\_1.a} = \text{Outc\_1.a}_{\text{sdized}}\ (\text{S1}|\text{S2})$$

Figure S6. Testimonials Pooled Analysis

		Outc_2.a S1	Outc_2.a S2	Outc_2.a	Outc_2.b S1	Outc_2.b S2	Outc_2.b
CONTROL	NA	X	X	Suppose that next month your household receives a grant of 7 lakis* which you can allocate between improved toilet, water and electricity. How many lakis would you allocate to toilet? ** 1 laki = $43* *➔ Number of lakis*	X	X	Of these, what is the most important way to keep children healthy? a. Have an improved toilet; b. Give them nutritious food; c. Have access to a health centre *➔ 1 = answered a.*
E3_T2 TESTIMONIAL #1	Devota lost her 3-month old baby Julius to dehydration following his infection from fecal matter. Since then, she supports improved toilets.	X			X		
E4_T1 TESTIMONIAL #2	Devota's 3-month old baby Julius has been safe from infections with fecal matter. She thanks having an improved toilet for that.		X			X	
E4_T2 TESTIMONIAL #3	Devota's 3-month old baby Julius died of dehydration following his infection with fecal matter. She blames his death on lack of improved toilet.		X			X	

Pool_Outc_Testim = Index_S1$_{\text{sdized}}$ (Outc_2.a$_{\text{sdized}}$, Outc_2.b$_{\text{sdized}}$) *OR* Index_S2$_{\text{sdized}}$ (Outc_2.a$_{\text{sdized}}$, Outc_2.b$_{\text{sdized}}$)
Pool_Outc_Testim_2.a = Outc_2.a$_{\text{sdized}}$ (S1|S2)
Pool_Outc_Testim_2.b = Outc_2.b$_{\text{sdized}}$ (S1|S2)

Step-by-step guide on study implementation

The research team collaborated with a polling firm to collect the SMS survey data and deliver the different experimental treatments, following several relevant steps:

1. We used the initial sampling frame covering phone users in Tanzania provided by the survey firm, which subscribes to lists of sim card users owned by Tanzania's network providers. Through a process called "users indexing", conducted at regular intervals alongside phone providers, the firm registers sim card users who are willing to be approached for surveys in the future, and collects demographic data about them. Those phone users eventually form the sample frame used to draw the sample.

2. From the sampling frame of phone users, the survey firm randomly drew a sample to receive an initial text message offering them to participate, using several stratifying variables (gender, rural, regions, and age categories, see Table S1) to achieve representativeness with the national population.

3. Because not all phone users who were approached agreed to respond, this initial text was sent to a greater number of phone users than the planned sample size, until the required number of respondents was secured for each stratum. In practice, this meant sending the initial text offering to participate to a larger number of individuals coming from demographics that are under-represented in a typical SMS survey (e.g., older individuals from rural areas). The initial text offering to participate continued to be sent until the eventual sample converged to having observable descriptive measures comparable to the national population.

4. As part of consent to participate in the survey, we provided an information sheet with additional details via a link shared by SMS. After consent was provided, respondents would proceed to the questions.

5. Each question was shared by SMS, and limited to 160 characters, inclusive of response options. Participants would respond to each question by sending a text at zero cost.

6. After a set of demographic and behavioral questions, respondents were randomized into one of the pre-prepared treatment groups.

7. Participants would then see a new prompt depending on their treatment assignment in the form of an SMS. For example, experiment 1 would show "Information: 'Washing hands regularly saves lives'" to the control group, "Soccer player said:

‘Washing hands regularly saves lives’” to the first endorsement treatment group, and “Tanzania’s Ministry of Health said: ‘Washing hands regularly saves lives’” to the second endorsement treatment group.

8. Following the experimental exposure to the treatment arm, a further set of SMS questions capturing the study outcomes of interest were asked.
9. Once these were captured, participants would move on to a second experiment following the same process. In this way, the two survey rounds each had two experiments capturing baseline and outcome variables within the same survey. Each of the experiments are illustrated in more detail, including the treatment assignments, survey round, and prompts used, in Figures S1 to S6.
10. Respondents who completed the SMS survey received airtime, sent to the phone number they used to take the survey. The amount of airtime was equivalent to USD 0.36. Providing this compensation is in line with local customs to show appreciation, and with past experience from the survey firm.
11. The data from the SMS surveys were then captured in a back-end database that could be downloaded and analyzed, after being de-identified (since names and geo-codes were never collected, this meant removing phone numbers from the database).
12. We then analyzed the data following the empirical approach explained in detail in the methods section of the paper.

Descriptive Statistics

Table S1. Sample Quota Targets

Region of Tanzania		Rural or Urban		Age Group		Male or Female	
Arusha	3,8%	Urban	40,0%	18-25 years	35,6%	Male	50,0%
Dar-Es-Salaam	12,6%	Rural	60,0%	26-35 years	25,3%	Female	50,0%
Dodoma	4,6%			36+ years	39,1%		
Geita	3,9%						
Iringa	2,1%						
Kagera	5,5%						
Katavi	1,3%						
Kigoma	4,7%						
Kilimanjaro	3,7%						
Lindi	1,9%						
Manyara	3,2%						
Mara	3,9%						
Mbeya	6,0%						
Morogoro	6,1%						
Mtwara	2,8%						
Mwanza	5,0%						
Njombe	1,6%						
Pwani	1,2%						
Rukwa	2,2%						
Ruvuma	3,1%						
Shinyanga	3,4%						
Simiyu	3,5%						
Singida	3,1%						
Tabora	5,1%						
Tanga	5,8%						

Table S2. Descriptive Statistics for Survey #1

	mean	sd	min	max
General				
Age (years)	30.31	9.14	18.00	86.00
Gender is female	0.42	0.49	0.00	1.00
Lives in rural area	0.48	0.50	0.00	1.00
Lives in Dar es Salaam	0.15	0.35	0.00	1.00
Education				
Never attended school	0.02	0.13	0.00	1.00
Highest edu level is primary school	0.26	0.44	0.00	1.00
Highest edu level is secondary	0.45	0.50	0.00	1.00
Highest edu level is higher education	0.23	0.42	0.00	1.00
Occupation				
Occupation is unemployed	0.13	0.34	0.00	1.00
Occupation is pupil or student	0.13	0.33	0.00	1.00
Occupation is farmer	0.40	0.49	0.00	1.00
Occupation is employee	0.18	0.38	0.00	1.00
Occupation is business owner	0.11	0.31	0.00	1.00
Observations	1116			

Table S3. Descriptive Statistics for Survey #2

	mean	sd	min	max
General				
Age (years)	32.10	10.14	18.00	100.00
Gender is female	0.47	0.50	0.00	1.00
Lives in rural area	0.54	0.50	0.00	1.00
Lives in Dar es Salaam	0.13	0.34	0.00	1.00
Education				
Never attended school	0.02	0.12	0.00	1.00
Highest edu level is primary school	0.28	0.45	0.00	1.00
Highest edu level is secondary	0.43	0.50	0.00	1.00
Highest edu level is higher education	0.23	0.42	0.00	1.00
Occupation				
Occupation is unemployed	0.13	0.34	0.00	1.00
Occupation is pupil or student	0.12	0.32	0.00	1.00
Occupation is farmer	0.39	0.49	0.00	1.00
Occupation is employee	0.17	0.38	0.00	1.00
Occupation is business owner	0.13	0.34	0.00	1.00
Observations	1122			

Balance Tables

Table S4. Balance Table Extract – Experiment 1

Variable	(1) Control Mean/SE	(2) Soccer Player Mean/SE	(3) Ministry of Health Mean/SE	T-test P-value (1)-(2)	(1)-(3)	(2)-(3)
Lives in capital	0.13<br>(0.02)	0.15<br>(0.02)	0.12<br>(0.02)	0.54	0.64	0.27
Age (in years)	31.11<br>(0.49)	30.31<br>(0.42)	30.93<br>(0.46)	0.21	0.78	0.32
Gender is female	0.46<br>(0.02)	0.43<br>(0.02)	0.44<br>(0.02)	0.42	0.57	0.81
Location rural	0.55<br>(0.02)	0.49<br>(0.02)	0.48<br>(0.02)	0.10*	0.06*	0.82
Never attended school	0.02<br>(0.01)	0.02<br>(0.01)	0.01<br>(0.00)	0.82	0.10	0.15
Highest edu level is primary school	0.30<br>(0.02)	0.26<br>(0.02)	0.29<br>(0.02)	0.30	0.77	0.45
Highest edu level is secondary	0.44<br>(0.02)	0.47<br>(0.02)	0.42<br>(0.02)	0.42	0.50	0.13
Highest edu level is higher education	0.19<br>(0.02)	0.22<br>(0.02)	0.24<br>(0.02)	0.32	0.13	0.57
Occupation is unemployed	0.13<br>(0.02)	0.13<br>(0.02)	0.12<br>(0.02)	0.95	0.50	0.53
Occupation is farmer	0.47<br>(0.02)	0.38<br>(0.02)	0.40<br>(0.02)	0.01**	0.03**	0.70
Occupation is pupil or student	0.11<br>(0.02)	0.14<br>(0.02)	0.12<br>(0.02)	0.30	0.76	0.47
Occupation is employee	0.13<br>(0.02)	0.18<br>(0.02)	0.20<br>(0.02)	0.10	0.02**	0.43
Occupation is business owner	0.09<br>(0.01)	0.12<br>(0.02)	0.11<br>(0.02)	0.12	0.28	0.64
N	401	450	428			
F-test of joint significance (F-stat)				1.13	1.19	0.76
F-test, number of observations				851	829	878

Notes: Values in t-tests are means diff, * 0.10 ** 0.05 *** 0.01.

Table S5. Balance Table Extract – Experiment 2

Variable	(1) Control Mean/SE	(2) Model Mean/SE	(3) Medical Commentator Mean/SE	T-test P-value (1)-(2)	(1)-(3)	(2)-(3)
Lives in capital	0.12	0.17	0.11	0.06*	0.63	0.02**
	(0.02)	(0.02)	(0.02)			
Age (in years)	32.84	31.68	31.76	0.09*	0.16	0.91
	(0.50)	(0.49)	(0.59)			
Gender is female	0.48	0.47	0.47	0.92	0.96	0.96
	(0.03)	(0.03)	(0.03)			
Location rural	0.54	0.56	0.53	0.50	0.75	0.33
	(0.03)	(0.03)	(0.03)			
Never attended school	0.01	0.02	0.01	0.58	0.98	0.61
	(0.01)	(0.01)	(0.01)			
Highest edu level is primary school	0.26	0.30	0.29	0.17	0.25	0.82
	(0.02)	(0.02)	(0.02)			
Highest edu level is secondary	0.45	0.44	0.40	0.65	0.14	0.30
	(0.03)	(0.03)	(0.03)			
Highest edu level is higher education	0.24	0.21	0.25	0.27	0.75	0.16
	(0.02)	(0.02)	(0.02)			
Occupation is unemployed	0.14	0.12	0.15	0.45	0.60	0.20
	(0.02)	(0.02)	(0.02)			
Occupation is farmer	0.39	0.39	0.40	0.90	0.72	0.63
	(0.03)	(0.02)	(0.03)			
Occupation is pupil or student	0.12	0.11	0.12	0.66	0.99	0.68
	(0.02)	(0.02)	(0.02)			
Occupation is employee	0.16	0.20	0.16	0.19	0.91	0.24
	(0.02)	(0.02)	(0.02)			
Occupation is business owner	0.14	0.14	0.11	0.76	0.12	0.21
	(0.02)	(0.02)	(0.02)			
N	374	381	367			
F-test of joint significance (F-stat)				1.30	0.64	1.40
F-test, number of observations				755	741	748

Notes: Values in t-tests are means diff, * 0.10 ** 0.05 *** 0.01.

Table S6. Balance Table Extract – Experiment 3

Variable	(1) Control Mean/SE	(2) Model Mean/SE	(3) Medical Commentator Mean/SE	T-test P-value (1)-(2)	(1)-(3)	(2)-(3)
Lives in capital	0.14	0.13	0.12	0.71	0.44	0.69
	(0.02)	(0.02)	(0.02)			
Age (in years)	31.12	31.15	30.02	0.96	0.07*	0.08*
	(0.46)	(0.48)	(0.41)			
Gender is female	0.46	0.41	0.46	0.11	0.93	0.09*
	(0.02)	(0.02)	(0.02)			
Location rural	0.49	0.49	0.53	0.96	0.29	0.26
	(0.02)	(0.02)	(0.02)			
Never attended school	0.01	0.02	0.01	0.08*	0.35	0.37
	(0.00)	(0.01)	(0.01)			
Highest edu level is primary school	0.29	0.30	0.26	0.57	0.34	0.12
	(0.02)	(0.02)	(0.02)			
Highest edu level is secondary	0.45	0.44	0.44	0.75	0.76	0.99
	(0.02)	(0.02)	(0.02)			
Highest edu level is higher education	0.23	0.19	0.24	0.11	0.60	0.03**
	(0.02)	(0.02)	(0.02)			
Occupation is unemployed	0.13	0.14	0.12	0.82	0.44	0.31
	(0.02)	(0.02)	(0.02)			
Occupation is farmer	0.40	0.40	0.44	0.99	0.21	0.20
	(0.02)	(0.02)	(0.02)			
Occupation is pupil or student	0.14	0.11	0.12	0.14	0.28	0.70
	(0.02)	(0.01)	(0.02)			
Occupation is employee	0.17	0.16	0.18	0.72	0.54	0.33
	(0.02)	(0.02)	(0.02)			
Occupation is business owner	0.11	0.12	0.09	0.58	0.36	0.13
	(0.02)	(0.02)	(0.01)			
J. Kaseja	0.37	0.38	0.31	0.81	0.06*	0.03**
	(0.02)	(0.02)	(0.02)			
Ministry of Health	0.35	0.33	0.32	0.55	0.45	0.86
	(0.02)	(0.02)	(0.02)			
N	406	448	425			
F-test of joint significance (F-stat)				0.92	1.44	1.82**
F-test, number of observations				854	831	873

Notes: Values in t-tests are means diff, * 0.10 ** 0.05 *** 0.01.

Table S7. Balance Table Extract – Experiment 4

Variable	(1) Control Mean/SE	(2) Testimonial #2 Mean/SE	(3) Testimonial #3 Mean/SE	T-test P-value (1)-(2)	(1)-(3)	(2)-(3)
Lives in capital	0.15	0.13	0.12	0.31	0.22	0.85
	(0.02)	(0.02)	(0.02)			
Age (in years)	32.09	32.07	32.13	0.97	0.96	0.93
	(0.54)	(0.55)	(0.49)			
Gender is female	0.50	0.43	0.49	0.06*	0.77	0.11
	(0.03)	(0.03)	(0.03)			
Location rural	0.56	0.55	0.52	0.63	0.27	0.53
	(0.03)	(0.03)	(0.03)			
Never attended school	0.01	0.02	0.02	0.73	0.78	0.95
	(0.01)	(0.01)	(0.01)			
Highest edu level is primary school	0.29	0.29	0.27	0.87	0.57	0.69
	(0.02)	(0.02)	(0.02)			
Highest edu level is secondary	0.42	0.42	0.46	0.99	0.26	0.27
	(0.03)	(0.03)	(0.03)			
Highest edu level is higher education	0.24	0.23	0.23	0.75	0.94	0.81
	(0.02)	(0.02)	(0.02)			
Occupation is unemployed	0.15	0.12	0.13	0.26	0.37	0.81
	(0.02)	(0.02)	(0.02)			
Occupation is farmer	0.38	0.42	0.38	0.37	0.97	0.35
	(0.03)	(0.03)	(0.02)			
Occupation is pupil or student	0.11	0.12	0.12	0.72	0.77	0.94
	(0.02)	(0.02)	(0.02)			
Occupation is employee	0.19	0.16	0.18	0.27	0.80	0.40
	(0.02)	(0.02)	(0.02)			
Occupation is business owner	0.13	0.12	0.13	0.60	0.96	0.57
	(0.02)	(0.02)	(0.02)			
Soccer player	0.32	0.34	0.36	0.57	0.23	0.53
	(0.02)	(0.02)	(0.02)			
Medical commentator	0.33	0.35	0.30	0.62	0.34	0.15
	(0.02)	(0.02)	(0.02)			
N	376	366	380			
F-test of joint significance (F-stat)				0.91	0.84	0.86
F-test, number of observations				742	756	746

Notes: Values in t-tests are means diff, * 0.10 ** 0.05 *** 0.01.

Table S8. Balance Table - All Endorsement Treatment Arms Pooled

Variable	(1) Control Mean/SE	(2) Pool Treat Mean/SE	T-test P-value (1)-(2)
Respondent's age in years	31.55	30.98	0.12
	(0.28)	(0.22)	
Gender is female	0.45	0.44	0.35
	(0.01)	(0.01)	
Urban status of respondent is rural	0.52	0.50	0.36
	(0.01)	(0.01)	
Region is Dar es Salaam	0.14	0.14	0.68
	(0.01)	(0.01)	
Never attended school	0.01	0.02	0.57
	(0.00)	(0.00)	
Highest edu level is primary school	0.27	0.28	0.48
	(0.01)	(0.01)	
Highest edu level is secondary	0.45	0.43	0.38
	(0.01)	(0.01)	
Highest edu level is higher education	0.22	0.22	0.92
	(0.01)	(0.01)	
Occupation is unemployed	0.14	0.13	0.71
	(0.01)	(0.01)	
Occupation is farmer	0.41	0.39	0.16
	(0.01)	(0.01)	
Occupation is pupil or student	0.13	0.12	0.53
	(0.01)	(0.01)	
Occupation is employee	0.16	0.18	0.07*
	(0.01)	(0.01)	
Occupation is business owner	0.12	0.12	0.76
	(0.01)	(0.01)	
N	1130	1962	
F-test of joint significance (F-stat)			1.00
F-test, number of observations			3092

Notes: Values in t-tests are means diff, * 0.10 ** 0.05 *** 0.01.

Table S9. Balance Table - All Testimonial Treatment Arms Pooled

Variable	(1) Control Mean/SE	(2) Pool Treat Mean/SE	T-test P-value (1)-(2)
Respondent's age in years	31.42	31.25	0.71
	(0.36)	(0.28)	
Gender is female	0.48	0.44	0.18
	(0.02)	(0.01)	
Urban status of respondent is rural	0.51	0.52	0.75
	(0.02)	(0.02)	
Region is Dar es Salaam	0.15	0.13	0.13
	(0.01)	(0.01)	
Never attended school	0.01	0.02	0.34
	(0.00)	(0.00)	
Highest edu level is primary school	0.28	0.26	0.34
	(0.02)	(0.01)	
Highest edu level is secondary	0.44	0.44	0.87
	(0.02)	(0.01)	
Highest edu level is higher education	0.24	0.24	0.88
	(0.02)	(0.01)	
Occupation is unemployed	0.14	0.12	0.16
	(0.01)	(0.01)	
Occupation is farmer	0.38	0.41	0.32
	(0.02)	(0.01)	
Occupation is pupil or student	0.13	0.12	0.72
	(0.01)	(0.01)	
Occupation is employee	0.18	0.18	0.83
	(0.01)	(0.01)	
Occupation is business owner	0.12	0.12	0.65
	(0.01)	(0.01)	
N	734	1106	
F-test of joint significance (F-stat)			0.91
F-test, number of observations			1840

Notes: Values in t-tests are means diff, * 0.10 ** 0.05 *** 0.01.

Additional Models

Table S10. Pooled Effects of Endorsements – Adding Controls

	Index of handwashing and sanitation							
	(1)	(2)	(3)	(4)	(5)	(6)	(7)	(8)
Pool, all endorsement arms	0.091**	0.088**						
	(0.020)	(0.023)						
Subpool, arms with celebrity endorsement			0.105***	0.098**				
			(0.010)	(0.014)				
Subpool, arms with non-expert celebrities					0.125***	0.118***		
					(0.003)	(0.005)		
Subpool, arms with soccer player							0.102*	0.097*
							(0.056)	(0.065)
Model							0.165**	0.155**
							(0.020)	(0.027)
Medical commentator					0.018	0.014	0.006	0.003
					(0.795)	(0.842)	(0.927)	(0.965)
Ministry of health			0.020	0.035	0.031	0.045	0.019	0.034
			(0.767)	(0.612)	(0.654)	(0.512)	(0.790)	(0.632)
Constant	-0.043	-0.191**	-0.052	-0.197**	-0.031	-0.173**	-0.019	-0.162*
	(0.287)	(0.027)	(0.203)	(0.023)	(0.479)	(0.050)	(0.679)	(0.070)
Age		✓		✓		✓		✓
Gender		✓		✓		✓		✓
Rural		✓		✓		✓		✓
Capital		✓		✓		✓		✓
No school		✓		✓		✓		✓
Primary school		✓		✓		✓		✓
Secondary school		✓		✓		✓		✓
Farmer		✓		✓		✓		✓
Employee		✓		✓		✓		✓
Observations	3092	3092	3092	3092	3092	3092	3092	3092

This model pools five treatment arms on endorsements conducted in 2021.
The index of handwashing and toilet (Pool_Outc_Endorse) is standardized (see Supplementary Figure S5).
Controls for wave and experiment not shown, base levels not shown.
* 0.10 ** 0.05 *** 0.01, p-val in parentheses.

Table S11. Pooled Effects of Testimonials – Adding Controls

	Index of Improved Sanitation			
	(1)	(2)	(3)	(4)
Pool, all testimonials arms	0.112**	0.106**		
	(0.028)	(0.037)		
Testimonial 1			0.147*	0.141*
			(0.062)	(0.073)
Testimonial 2			0.057	0.049
			(0.462)	(0.531)
Testimonial 3			0.116	0.113
			(0.130)	(0.142)
Constant	-0.017	0.034	-0.000	0.051
	(0.719)	(0.767)	(1.000)	(0.663)
Age		✓		✓
Gender		✓		✓
Rural		✓		✓
Capital		✓		✓
No school		✓		✓
Primary school		✓		✓
Secondary school		✓		✓
Farmer		✓		✓
Employee		✓		✓
Observations	1840	1840	1840	1840

This model pools three treatment arms on testimonials conducted in 2021.
The index of improved sanitation (Pool_Outc_Testim) is standardized (see Supplementary Figure S6).
Controls for wave and experiment not shown, base levels not shown.
* 0.10 ** 0.05 *** 0.01, p-val in parentheses.

Table S12. Pooled Effects of Endorsements – Adding Individuals Surveyed Several Times

	Index of handwashing and sanitation				Water use (liters)			
	(1)	(2)	(3)	(4)	(5)	(6)	(7)	(8)
Pool, all endorsement arms	0.100***				0.073			
	(0.009)				(0.104)			
Subpool, arms with celebrity endorsement		0.105***				0.078		
		(0.008)				(0.106)		
Subpool, arms with non-expert celebrities			0.125***				0.103**	
			(0.003)				(0.045)	
Subpool, arms with soccer player				0.103**				0.103**
				(0.045)				(0.045)
Model				0.165**				
				(0.020)				
Medical commentator			0.018	0.007			0.007	0.007
			(0.795)	(0.919)			(0.919)	(0.919)
Ministry of health		0.074	0.085	0.073		0.060	0.073	0.073
		(0.251)	(0.194)	(0.274)		(0.367)	(0.274)	(0.274)
Constant	-0.049	-0.053	-0.031	-0.020	-0.031	-0.034	-0.020	-0.020
	(0.222)	(0.196)	(0.478)	(0.665)	(0.469)	(0.441)	(0.665)	(0.665)
Observations	3255	3255	3255	3255	2401	2401	2401	2401

This model pools five treatment arms on endorsements conducted in 2021.
The index of handwashing and toilet (Pool_Outc_Endorse) is standardized (see Supplementary Figure S5).
Water use (Pool_Outc_1.a) pools information across experiments and is standardized (see Supplementary Figure S5).
Controls for wave and experiment not shown, base levels not shown * 0.10 ** 0.05 *** 0.01, p-val in parentheses,
Duplicate observations left in.

Table S13. Pooled Effects of Testimonials – Adding Individuals Surveyed Several Times

	Index of improved sanitation		Sanitation investment		Sanitation priority	
	(1)	(2)	(3)	(4)	(5)	(6)
Pool, all testimonials arms	0.107**		-0.008		0.144***	
	(0.032)		(0.865)		(0.004)	
Testimonial #1		0.130*		0.020		0.149**
		(0.078)		(0.771)		(0.043)
Testimonial #2		0.057		0.019		0.057
		(0.464)		(0.793)		(0.467)
Testimonial #3		0.116		-0.081		0.219***
		(0.132)		(0.271)		(0.005)
Constant	-0.013	-0.000	-0.016	0.000	-0.003	0.000
	(0.778)	(1.000)	(0.718)	(1.000)	(0.949)	(1.000)
Observations	1953	1953	1953	1953	1953	1953

This model pools three treatment arms on testimonials conducted in 2021.
Sanitation investment (Pool_Outc_2.a) pools information across experiments and is standardized (see Supplementary Figure S6).
Sanitation priority (Pool_Outc_2.b) pools information across experiments and is standardized (see Supplementary Figure S6).
Controls for wave and experiment not shown, base levels not shown * 0.10 ** 0.05 *** 0.01, p-val in parentheses.
Duplicate observations left in.

Table S14. Pooled Effects of Endorsements – Adding Regional Fixed Effects

	Index of handwashing and sanitation							
	(1)	(2)	(3)	(4)	(5)	(6)	(7)	(8)
Pool, all endorsement arms	0.091**	0.088**						
	(0.020)	(0.024)						
Subpool, arms with celebrity endorsement			0.105***	0.100**				
			(0.010)	(0.013)				
Subpool, arms with non-expert celebrities					0.125***	0.120***		
					(0.003)	(0.005)		
Subpool, arms with soccer player							0.102*	0.099*
							(0.056)	(0.063)
Model							0.165**	0.157**
							(0.020)	(0.026)
Medical commentator					0.018	0.016	0.006	0.006
					(0.795)	(0.813)	(0.927)	(0.936)
Ministry of health			0.020	0.023	0.031	0.034	0.019	0.022
			(0.767)	(0.734)	(0.654)	(0.629)	(0.790)	(0.754)
Constant	-0.043	-0.248***	-0.052	-0.255***	-0.031	-0.233**	-0.019	-0.222**
	(0.287)	(0.009)	(0.203)	(0.008)	(0.479)	(0.016)	(0.679)	(0.023)
Regional FE		✓		✓		✓		✓
Observations	3092	3092	3092	3092	3092	3092	3092	3092

This model pools five treatment arms on endorsements conducted in 2021.
The index of handwashing and sanitation (Pool_Outc_Endorse) is standardized (see Supplementary Figure S5).
Controls for wave and experiment not shown, base levels not shown * 0.10 ** 0.05 *** 0.01, p-val in parentheses.

Table S15. Pooled Effects of Testimonials – Adding Regional Fixed Effects

	Index of Improved Sanitation			
	(1)	(2)	(3)	(4)
Pool, all testimonials arms	0.112**	0.112**		
	(0.028)	(0.027)		
Testimonial 1			0.147*	0.137*
			(0.062)	(0.081)
Testimonial 2			0.057	0.064
			(0.462)	(0.405)
Testimonial 3			0.116	0.124
			(0.130)	(0.106)
Constant	-0.017	-0.400***	-0.000	-0.386***
	(0.719)	(0.001)	(1.000)	(0.002)
Regional FE		✓		✓
Observations	1840	1840	1840	1840

This model pools three treatment arms on testimonials conducted in 2021.
The index of improved sanitation (Pool_Outc_Testim) is standardized (see Supplementary Figure S6).
Controls for wave and experiment not shown, base levels not shown.
* 0.10 ** 0.05 *** 0.01, p-val in parentheses.

Table S16. Pooled Effects of Endorsements – Standard Errors Clustered by Individual Respondent

	Index of handwashing and sanitation				Water use (liters)			
	(1)	(2)	(3)	(4)	(5)	(6)	(7)	(8)
Pool, all endorsement arms	0.091**				0.059			
	(0.017)				(0.202)			
Subpool, arms with celebrity endorsement		0.105***				0.075		
		(0.009)				(0.123)		
Subpool, arms with non-expert celebrities			0.125***				0.102*	
			(0.003)				(0.055)	
Subpool, arms with soccer player				0.102*				0.102*
				(0.056)				(0.055)
Model				0.165**				
				(0.020)				
Medical commentator			0.018	0.006			0.006	0.006
			(0.791)	(0.926)			(0.926)	(0.926)
Ministry of health		0.020	0.031	0.019		0.005	0.019	0.019
		(0.767)	(0.653)	(0.790)		(0.946)	(0.790)	(0.790)
Constant	-0.043	-0.052	-0.031	-0.019	-0.021	-0.032	-0.019	-0.019
	(0.277)	(0.194)	(0.473)	(0.672)	(0.617)	(0.461)	(0.672)	(0.672)
Observations	3092	3092	3092	3092	2238	2238	2238	2238

See notes of Table 1.

Table S17. Pooled Effects of Testimonials – Standard Errors Clustered by Individual Respondent

	Index of Improved Sanitation		Sanitation Investment		Sanitation priority	
	(1)	(2)	(3)	(4)	(5)	(6)
Pool, all testimonials arms	0.112**		0.000		0.145***	
	(0.024)		(0.994)		(0.004)	
Testimonial 1		0.147*		0.045		0.154**
		(0.054)		(0.541)		(0.044)
Testimonial 2		0.057		0.019		0.057
		(0.451)		(0.796)		(0.451)
Testimonial 3		0.116		-0.081		0.219***
		(0.140)		(0.271)		(0.006)
Constant	-0.017	-0.000	-0.021	0.000	-0.004	0.000
	(0.708)	(1.000)	(0.627)	(1.000)	(0.931)	(1.000)
Observations	1840	1840	1840	1840	1840	1840

See notes of Table 2
